# Antioxidant effect of nicotinamide mononucleotide in tendinopathy

**DOI:** 10.1186/s12891-022-05205-z

**Published:** 2022-03-14

**Authors:** Kohei Yamaura, Yutaka Mifune, Atsuyuki Inui, Hanako Nishimoto, Takashi Kurosawa, Shintaro Mukohara, Yuichi Hoshino, Takahiro Niikura, Ryosuke Kuroda

**Affiliations:** Department of Orthopaedic Surgery, Kobe University Graduate School of Medicine, 7-5-1, Kusunoki-cho, Chuo-ku, Kobe, 650-0017 Japan

**Keywords:** Nicotinamide mononucleotide, Oxidative stress, SIRT, Tendinopathy

## Abstract

**Background:**

A link between tendinopathy and oxidative stress has been recently reported. Nicotinamide mononucleotide (NMN) is a precursor of nicotinamide adenine dinucleotide, which plays an important role in cell redox homeostasis. The aim of this study was to evaluate the antioxidant effect of NMN on tendinopathy in vitro and in vivo.

**Methods:**

Tenocytes from healthy Sprague-Dawley rats were cultured in regular glucose (RG) and high-glucose (HG) conditions with or without NMN, and were divided into four groups: RG NMN(−), RG NMN(+), HG NMN(−), and HG NMN(+). Cell viability, reactive oxygen species (ROS) accumulation, apoptotic rate, and mRNA expression of nicotinamide adenine dinucleotide phosphate oxidase (NOX)1, NOX4, interleukin (IL)6, sirtuin (SIRT)1, and SIRT6 were investigated. In addition, rats with collagenase-induced tendinopathy were treated with or without NMN. Immunostaining of NOX1 and NOX4; mRNA expression of SIRT1, SIRT6, and IL6; and superoxide dismutase (SOD) activity measurements in the Achilles tendon were performed.

**Results:**

NMN increased the expression of SIRT1 and SIRT6 in rat tenocytes, but decreased the levels of NOX1, NOX4, IL6, ROS, and apoptosis. In Achilles tendons with collagenase-induced tendinopathy, NMN increased the mRNA expression of SIRT1 and SIRT6, as well as SOD activity; while suppressing protein expression of NOX1 and NOX4, and mRNA expression of IL6.

**Conclusion:**

The in vitro and in vivo results of this study show that NMN exerts an antioxidant effect on tendinopathy by promoting the expression of SIRT while inhibiting that of NOX.

## Background

Tendinopathy is a common and debilitating musculoskeletal disorder caused by injury or degeneration of tendons, and which accounts for more than 30% of musculoskeletal lesions [1, 2]. Tendinopathy is characterized by pain arising from repetitive motion and localized tenderness, which leads to decreased productivity and physical activity [3]. Despite the high prevalence of tendinopathy, its etiology and pathogenesis remain poorly understood, with existing treatments focusing mainly on the relief of symptoms. Tendinopathy originates primarily from overload, repetitive injury, and inflammation caused by microtrauma, and becomes obvious when tissue destruction exceeds the tendon’s healing capacity [1, 4, 5]. Impaired tendon repair, in the process of tendinopathy, leads to scarring and fibrosis, which reduces mechanical strength and increases the risk of secondary damage [1, 5, 6]. Inadequate healing of the injured tendon is related to low blood supply and reduced metabolic activity of resident cells, which impairs tissue homeostasis [7, 8]. Recently, the involvement of oxidative stress and apoptosis in the pathogenesis of tendinopathy has been proposed [9–11]. Oxidative stress is caused mainly by reactive oxygen species (ROS), which regulate various signaling pathways and cellular functions [12, 13]. Overproduction of ROS can damage proteins, DNA, and RNA, causing tissue, cell, and organ dysfunction [14]. A previous study reported that the main source of ROS was nicotinamide adenine dinucleotide phosphate oxidase (NOX) [15]. As we have previously shown, inhibition of NOX suppressed hyperglycemia-induced overproduction of ROS in rat tenocytes [16].

Nicotinamide adenine dinucleotide (NAD^+^) plays an important role in energy metabolism, aging, and cell death, with its levels decreasing with age [17, 18]. Nicotinamide mononucleotide (NMN) is a precursor of NAD^+^, to which it is converted by nicotinamide mononucleotide adenylyltransferase [18]. NMN has been shown to suppress age-related deterioration of various organs in animal models [19, 20], as well as inhibit oxidative stress and apoptosis [21, 22]. However, no studies have assessed the antioxidant potential of NMN in tenocytes.

In the present study, we hypothesized that NMN could inhibit the excessive oxidative stress in tendinopathy. Thus, the aim of the study was to evaluate the antioxidant effect of NMN on tendinopathy in vitro and in vivo. To investigate the effect in vitro, the rat tenocyte oxidative stress model induced by hyperglycemia was used [11, 16]. To investigate the effect in vivo, a collagenase-induced rat tendinopathy model similar to that of human tendinopathy was employed [8].

## Material and methods

All animal procedures were performed following the approved experimental protocol and the guidelines of the Animal Care and Use Committee of our institution.

### In vitro experiments

#### Cell culture

Eight 8-week-old Sprague-Dawley female rats purchased from Japan SLC (Shizuoka, Japan) were used for the in vitro experiments. After the rats were euthanized by inhalation of isoflurane (Wako Pure Chemical Corp., Osaka, Japan) and an overdose of sodium pentobarbital (200 mg/kg; Kyoritsu Seiyaku, Tokyo, Japan) administered by intraperitoneal injection, the Achilles tendons were excised. The tendons were washed with phosphate-buffered saline (PBS) and cut into small pieces of approximately 1.0–2.0 mm^3^. Subsequently, several pieces of the tendon were placed on a culture plate and cultured in Dulbecco’s modified Eagle’s medium (DMEM; HyClone, Logan, UT, USA) supplemented with 10% fetal bovine serum (Cansera, Rexdale, ON, Canada), 100 U/mL penicillin, and 100 μg/mL streptomycin at 37 °C in a humidified atmosphere of 5% CO_2_. The cells obtained from the tendons were subcultured after trypsin digestion and tenocytes at passage 2 were used for subsequent experiments.

#### Cell proliferation assay

Cell proliferation was evaluated by a water-soluble tetrazolium salt (WST) assay using the Cell Counting Kit-8 (Dojindo, Kumamoto, Japan) [23]. Each well in 96-well plates was filled with 100 μL DMEM containing 6 mM glucose and 2 × 10^4^ cells. The wells were divided into four different samples containing 0, 0.01, 0.1, or 1 mM NMN (Oriental Yeast Co., Tokyo, Japan). The plates were placed in a CO_2_ incubator at 37 °C for 48 h, after which 10 μL of WST was added to each well and the plates were incubated for 4 h at 37 °C. The conversion of WST to formazan was measured at 450 nm using a spectrophotometer (*n* = 4 per group).

#### Experimental protocol

**Fig. 1:**
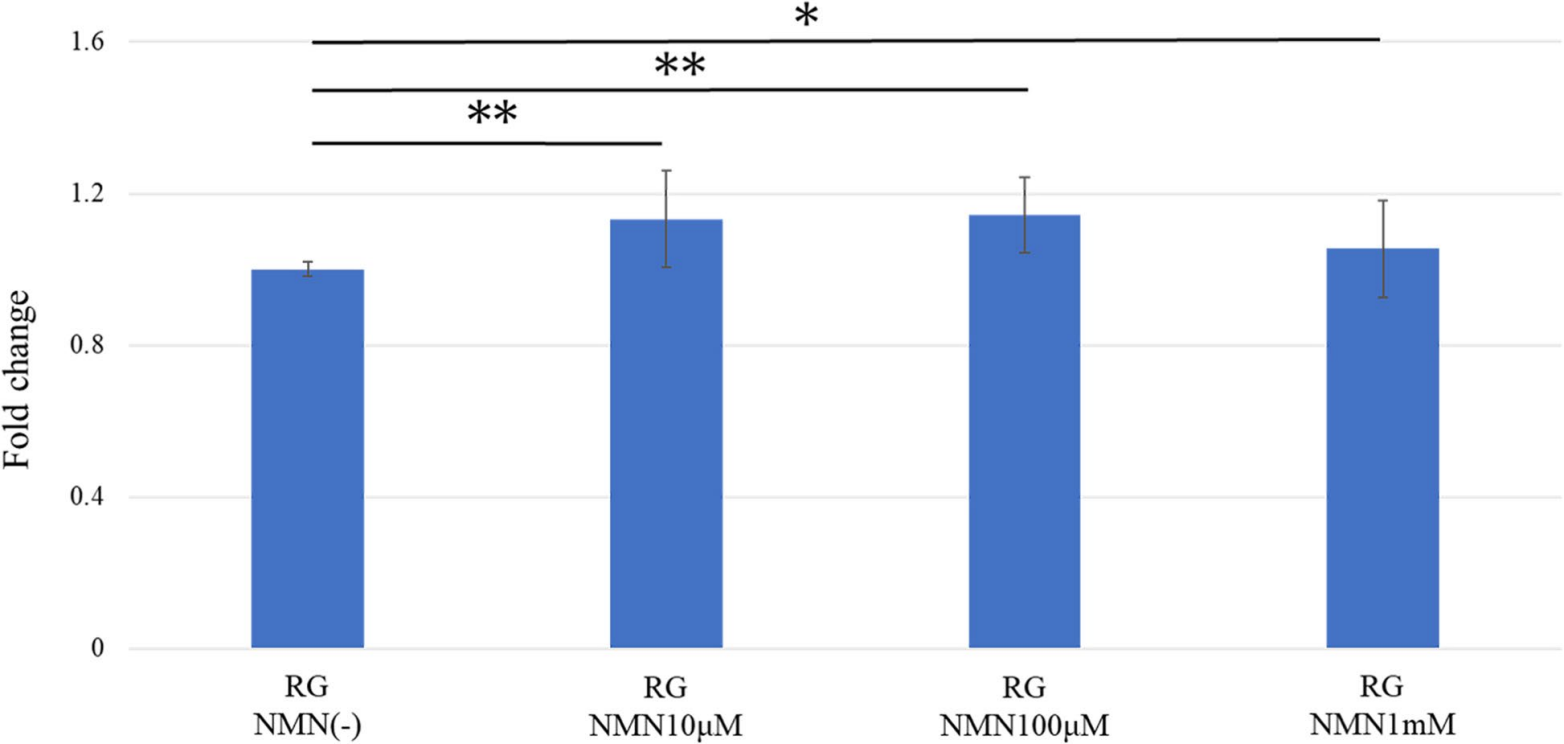
Cell proliferation in the presence of NMN. Proliferation of tenocytes treated with different concentrations of NMN. Cell proliferation was significantly higher in NMN-treated groups than in the untreated control group. * *p* < 0.05; ** *p* < 0.0001

Each well in 12-well plates was seeded with 1 × 10^5^ tenocytes. The tenocytes were incubated in DMEM at two different glucose concentrations: 6 mM in the regular-glucose (RG) group, and 33 mM in the high-glucose (HG) group. NMN was dissolved in PBS to a final concentration of 0.1 mM based on the outcome of the cell proliferation assay (Fig. 1). Tenocytes were divided into four groups: a) RG group without NMN (RG NMN(−)), b) RG group with NMN (RG NMN(+)), c) HG group without NMN (HG NMN(−)), and d) HG group with NMN (HG NMN(+)).

#### Quantitative reverse transcription PCR (RT-PCR) analysis

Total RNA from tenocytes was extracted using a RNeasy Mini Kit (Qiagen, Valencia, CA, USA) after cell cultivation for 48 h. Total RNA was reverse transcribed to cDNA using a high-capacity cDNA reverse transcription kit (Applied Biosystems, Foster City, CA, USA). The cDNA was then amplified in triplicate in an Applied Biosystems 7900HT fast real-time PCR system using SYBR Green reagents (Applied Biosystems). Housekeeping gene expression was used for normalization and test genes were compared to the control (untreated) culture using the 2^−ΔΔCt^ method (*n* = 8 per group). Primer pair sequences are listed in Table 1.

**Table 1:** Primer sequences used for the polymerase chain reaction

Gene	Oligonucleotide sequence
NOX1	Forward 5′ GTGGCTTTGGTTCTCATGGT 3′ Reverse 5′ TGAGGACTCCTGCAACTCCT 3′
NOX4	Forward 5′ GGGCCTAGGATTGTGTTTGA 3′ Reverse 5′ CTGAGAAGTTCAGGGCGTTC 3′
IL6	Forward 5′ GGTCTTCTGGAGTTCCGTTTC 3′ Reverse 5′ GGTCTTGGTCCTTAGCCACTC 3′
SIRT1	Forward 5′ GACGACGAGGGCGAGGAG 3′ Reverse 5′ ACAGGAGGTTGTCTCGGTAGC 3′
SIRT6	Forward 5′ GCCGTCTGGTCATTGTCA 3′ Reverse 5′ AGCCTTGGGTGCTACTGG 3′
GAPDH	Forward5′ GGTGGTCTCCTCTGACTTCAACA 3′ Reverse 5′ GTTGCTGTAGCCAAATTCGTTGT 3′

#### ROS measurements

The accumulation of intracellular ROS in tenocytes grown for 48 h was detected using the oxidation-sensitive fluorescent probe 2′7′-dichlorofluorescin diacetate supplied in the total ROS/Superoxide detection kit (Enzo Life Sciences, Farmingdale, NY, USA). Each well of a 12-well plate containing tenocytes was filled with the fluorescent probe at a final concentration of 10 μM, and the plate was incubated for 60 min at 37 °C in the dark. Subsequently, the tenocytes were washed three times with PBS, trypsinized, and resuspended. To quantify ROS accumulation, the number of ROS-positive cells and 2-(4-amidinophenyl)-1H-indole-6-carboxamidine (DAPI)-positive cells in four randomly selected areas on each slide was counted by two independent investigators, and the mean value of each was calculated. The percentage of ROS-positive cells was calculated using the formula (number of ROS-positive nuclei/number of DAPI-positive nuclei) × 100 (*n* = 8 per group).

#### Immunofluorescence staining of apoptotic cells

Nuclear fragmentation was detected by TUNEL staining with an APO-DIRECT™ kit (Phoenix Flow Systems, San Diego, CA, USA) using fixed cells (4% paraformaldehyde/PBS) and DAPI. For quantitative analysis of apoptosis, the number of apoptotic cells and DAPI-positive cells in four randomly selected areas on each slide was counted by two independent investigators, and the mean value of each was calculated. The percentage of apoptotic cells was calculated using the following formula: (number of apoptosis-positive cells/number of DAPI-positive cells) × 100 (*n* = 8 per group).

### In vivo experiments

#### Creation of a collagenase-induced tendinopathy rat model

Eight 8-week-old female Sprague-Dawley rats (mean body weight, 212 ± 9 g) purchased from Japan SLC were used for the in vivo experiments. The rats were anesthetized with isoflurane and intraperitoneal injection of 0.15 mg/kg medetomidine (Kyoritsu Seiyaku Corp.), 2 mg/kg midazolam (Astellas Pharma Inc., Tokyo, Japan), and 2.5 mg/kg butorphanol (Meiji Seika Pharma Co., Ltd., Tokyo, Japan). After disinfection, the Achilles tendon was palpated under aseptic conditions and a 0.5-cm longitudinal skin incision was made slightly medial to its center to bluntly detach and expose the tendon. Subsequently, the rats were injected in the middle of bilateral Achilles tendons with a 30G needle to deliver 30 μL type I collagenase (3.0 mg/mL; Wako Pure Chemical Corp) dissolved in PBS [8]. Finally, the skin was sutured using 4–0 nylon. Postoperatively, each rat was kept separately and allowed to be active in the cage without restriction.

#### Experimental protocol

The rats from the collagenase-induced tendinopathy model described above were randomly divided into two groups: the control group and the NMN group (*n* = 4 per group). The acute phase of collagenase-induced tendinopathy in rats is 3–15 days after injection, and the acute tendinopathy model is established approximately 2 weeks after collagenase injection [8]. Therefore, NMN or PBS was administered 2 weeks after injection. In the NMN group, 1 mL of NMN solution equivalent to 100 mg/kg of NMN was injected intraperitoneally every other day for 2 weeks [20, 24–26]. The control group was injected with 1 mL PBS in the same way. The rats were euthanized by inhalation of isoflurane and an overdose of sodium pentobarbital (200 mg/kg; Kyoritsu Seiyaku Corp.) administered by intraperitoneal injection 2 weeks after treatment with NMN or PBS. The sample size for the in vivo experiments was calculated based on previous studies [27], using a priori power analysis (α < 0.05, 1-β ≥ 0.8) in G*Power (v 3.1; Düsseldorf University), and the sample size required for each group in the unpaired *t*-test was *n* = 4. The number of rats to be sacrificed was reduced by performing collagenase injections into the bilateral Achilles tendons. Given that four rats injected in the right Achilles tendon were used for immunostaining and four injected in the left Achilles tendon were used for PCR and superoxide dismutase (SOD) activity evaluation, the total number of rats required for the in vivo study was limited to 8 (*n* = 4 per group).

#### NOX immunostaining

Four Achilles tendons of rats in each group were used for immunohistological analysis of NOX1 and NOX4. Longitudinal sections of frozen Achilles tendons were cut to a thickness of 7 μm and fixed with 10% phosphate-buffered paraformaldehyde at room temperature. The sections were incubated with proteinase for 10 min and treated with 3% hydrogen peroxide (Wako Pure Chemical Corp.) to block endogenous peroxidase activity, and then incubated with anti-NOX1 or anti-NOX4 antibodies (both 1:1000; Abcam, Cambridge, UK) overnight at 4 °C. Sections were incubated with a peroxidase-labeled secondary antibody (Nichirei Bioscience, Tokyo, Japan) for 30 min at room temperature, followed by incubation with 3,3′-diaminobenzidine peroxidase substrate (Nichirei Bioscience) [11, 27]. Sections were counterstained with hematoxylin. To quantitatively evaluate NOX1 and NOX4 immunostaining, the percentage of NOX1 and NOX4 staining was calculated by two independent researchers using Adobe Photoshop CC 2020 software (Adobe Systems Inc., San Jose, CA, USA) as the ratio of stained brown pixels to total pixels of the entire tendon in a randomly selected area from each tendon slide.

#### SOD activity assessment

SOD is a key antioxidant enzyme that detoxifies ROS in vivo [28]. SOD activity can be measured as the 50% inhibition of a colorimetric reaction between a WST substrate and the superoxide anion [29], thus indicating the amount of ROS that can be removed [30]. In this study, the SOD activity of four Achilles tendons in each group was measured using a SOD Assay Kit-WST (Dojin Chemicals Co., Kumamoto, Japan) according to the manufacturer’s protocol. Tendon tissues were weighed wet, homogenized with sucrose buffer (0.25 M sucrose, 10 mM Tris-HCl pH 7.4, 1 mM EDTA), centrifuged at 10,000×*g* for 60 min, and the supernatant was retained. Absorbance at 450 nm was measured after incubation at 37 °C for 20 min according to the manufacturer’s protocol. SOD activity per wet weight of the tendon was calculated by determining the ROS inhibition rate % and the concentration of SOD causing 50% inhibition (U/mL).

#### Quantitative RT-PCR analysis

For quantitative RT-PCR, four Achilles tendons from each group were used. The Achilles tendons were cut into small pieces, enzymatically dissociated with type II collagenase (Worthington Biochemical Corp., Lakewood, NJ, USA), and total RNA was extracted using the RNeasy Mini Kit. Reverse transcription to single-stranded cDNA and real-time PCR were performed in the same way as with in vitro samples, and the expression of interleukin (IL)6, sirtuin (SIRT)1, and SIRT6 was evaluated.

#### Statistical analyses

Statistical analyses were performed using SPSS software (version 18.0; SPSS Inc., Chicago, IL, USA), and the data were expressed as the mean ± standard deviation. Comparisons between more than two groups were performed using two-way ANOVA and Tukey’s post-hoc test. Comparisons between two groups were performed using the Mann-Whitney test. Statistical significance was set at *p* < 0.05.

## Results

### In vitro experiments

#### Cell proliferation assay

Cell proliferation was significantly higher in NMN-treated groups than in the untreated control group (Fig. 1). Each *p*-values compared to the control group was *p* < 0.0001, *p* < 0.0001, and *p* = 0.048, in NMN 10, 100, 1 mM groups, respectively. Proliferation was highest in the 0.1 mM NMN group, although the difference was not significant compared to the other two doses of NMN. Therefore, 0.1 mM was chosen as the NMN concentration for subsequent experiments.

#### Gene expression analysis by RT-PCR

**Fig. 2:**
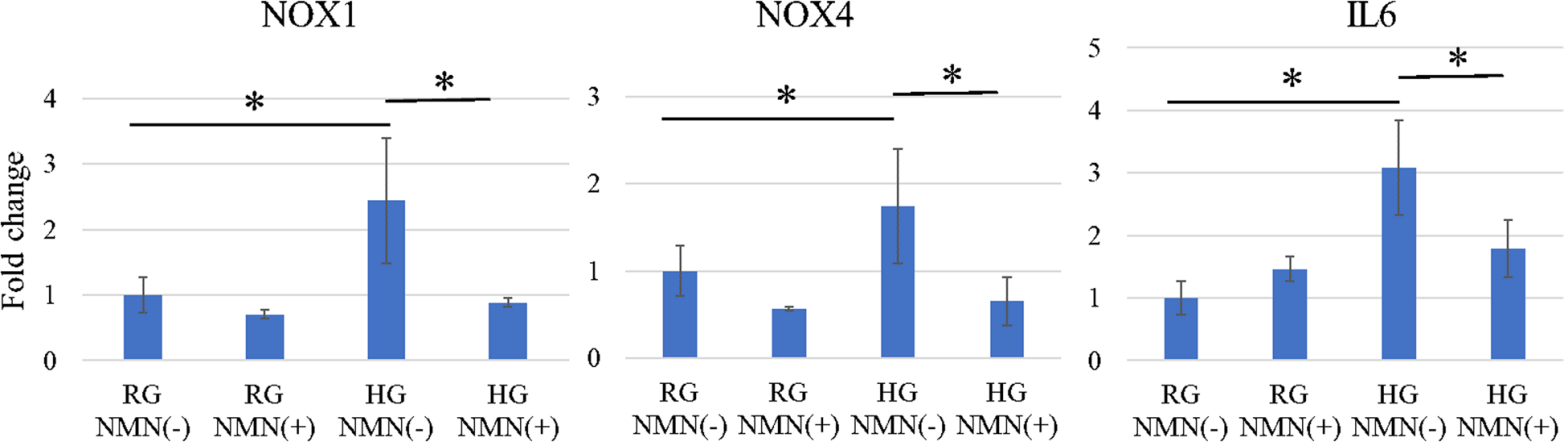
Quantitative expression of NOX1, NOX4, and IL6 mRNA in vitro. Quantitative RT-PCR results showing mRNA expression of NOX1, NOX4, and IL6 in four different in vitro conditions: regular-glucose without NMN (RG NMN(−)), regular-glucose with NMN (RG NMN(+)), high-glucose without NMN (HG NMN(−)), and high-glucose with NMN (HG NMN(+)). The mRNA expression of NOX1, NOX4, and IL6 was significantly higher in the HG NMN(−) group than in the RG NMN(−) and HG NMN(+) groups at 48 h. *p < 0.05

**Fig. 3:**
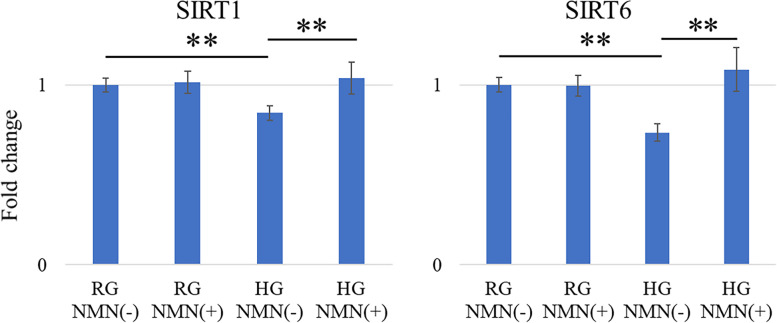
Quantitative expression of SIRT1 and SIRT6 mRNA in vitro. Quantitative RT-PCR results showing mRNA expression of SIRT1 and SIRT6 in four different in vitro conditions: regular-glucose without NMN (RG NMN(−)), regular-glucose with NMN (RG NMN(+)), high-glucose without NMN (HG NMN(−)), and high-glucose with NMN (HG NMN(+)) The mRNA expression of SIRT1 and SIRT6 was significantly higher in the RG NMN(−) and HG NMN(+) groups than in the HG NMN(−) group. **p < 0.0001

The mRNA expression of NOX1, NOX4, and IL6 was significantly higher in the HG NMN(−) group than in the RG NMN(−) group (*p* = 0.047, *p* = 0.043, and *p* = 0.0017, respectively) and HG NMN(+) group (*p* = 0.034, *p* = 0.0084, and *p* = 0.044, respectively) at 48 h (Fig. 2). Conversely, the mRNA expression of SIRT1 and SIRT6 was significantly higher in the RG NMN(−) group (both; *p* < 0.0001) and HG NMN(+) group (both; *p* < 0.0001) than in the HG NMN(−) group (Fig. 3).

#### ROS accumulation in tenocytes

**Fig. 4:**
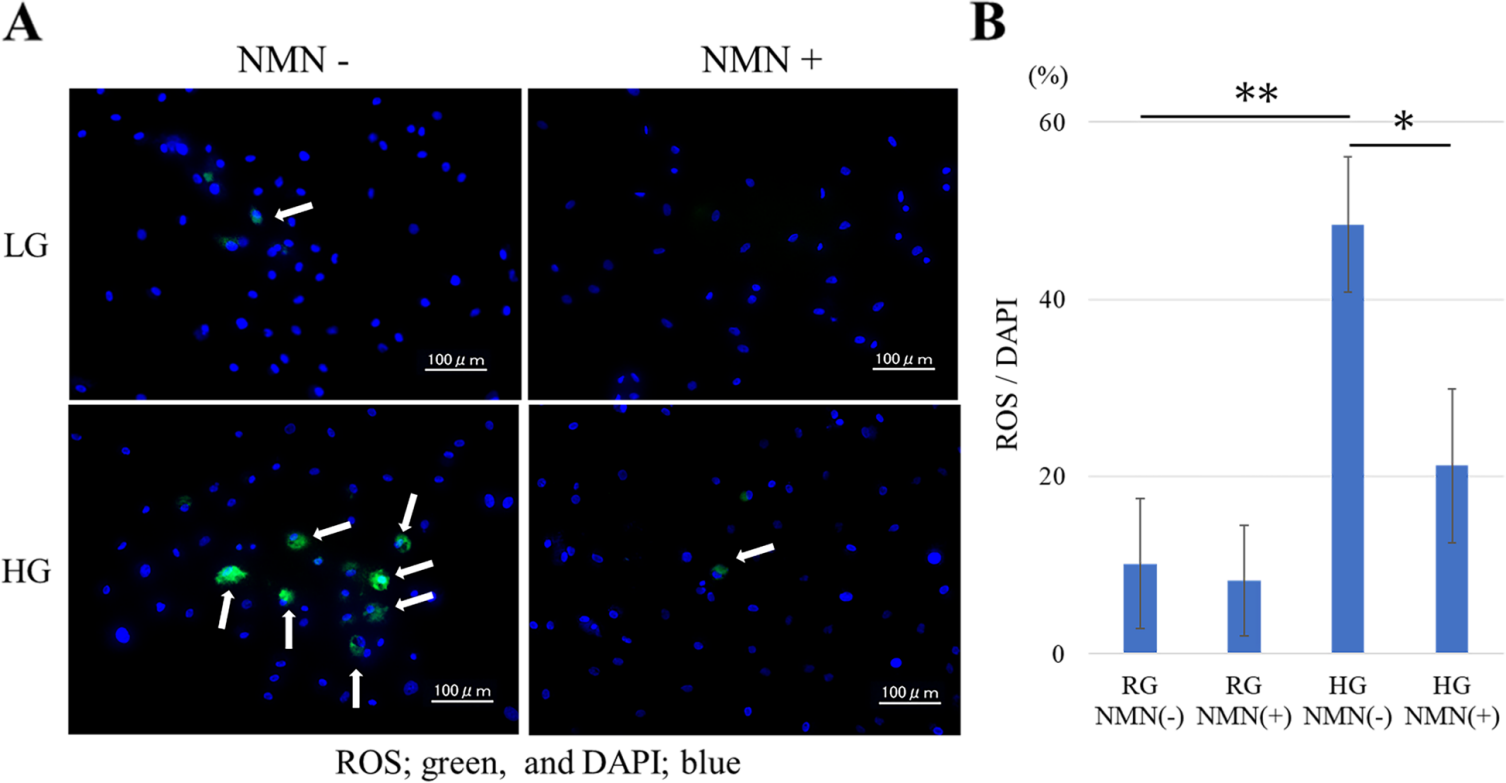
ROS accumulation in vitro. Analysis of ROS accumulation in four different in vitro conditions: regular-glucose without NMN (RG NMN(−)), regular-glucose with NMN (RG NMN(+)), high-glucose without NMN (HG NMN(−)), and high-glucose with NMN (HG NMN(+)). (**a**) Fluorescence staining showing ROS accumulation (green; white arrow) and DAPI staining (blue) in tenocytes. (**b**) Quantification of intracellular ROS levels in the four groups. ROS accumulation was significantly higher in the HG NMN(−) group than in the RG NMN(−) group, whereas that was significantly lower in the HG NMN(+) group than in the HG NMN(−) group. * *p* < 0.05; ** *p* < 0.0001

Fluorescence staining revealed ROS accumulation in tenocytes (Fig. 4A). Based on counterstaining with DAPI, the accumulation of intracellular ROS was significantly higher in the HG NMN(−) group than in the RG NMN(−) group at 48 h (*p* < 0.0001) (Fig. 4B). Importantly, though, ROS accumulation was significantly lower in the HG NMN(+) group than in the HG NMN(−) group (*p* = 0.008); whereas no significant difference was detected within the RG groups (Fig. 4B).

#### Analysis of cell apoptosis

**Fig. 5:**
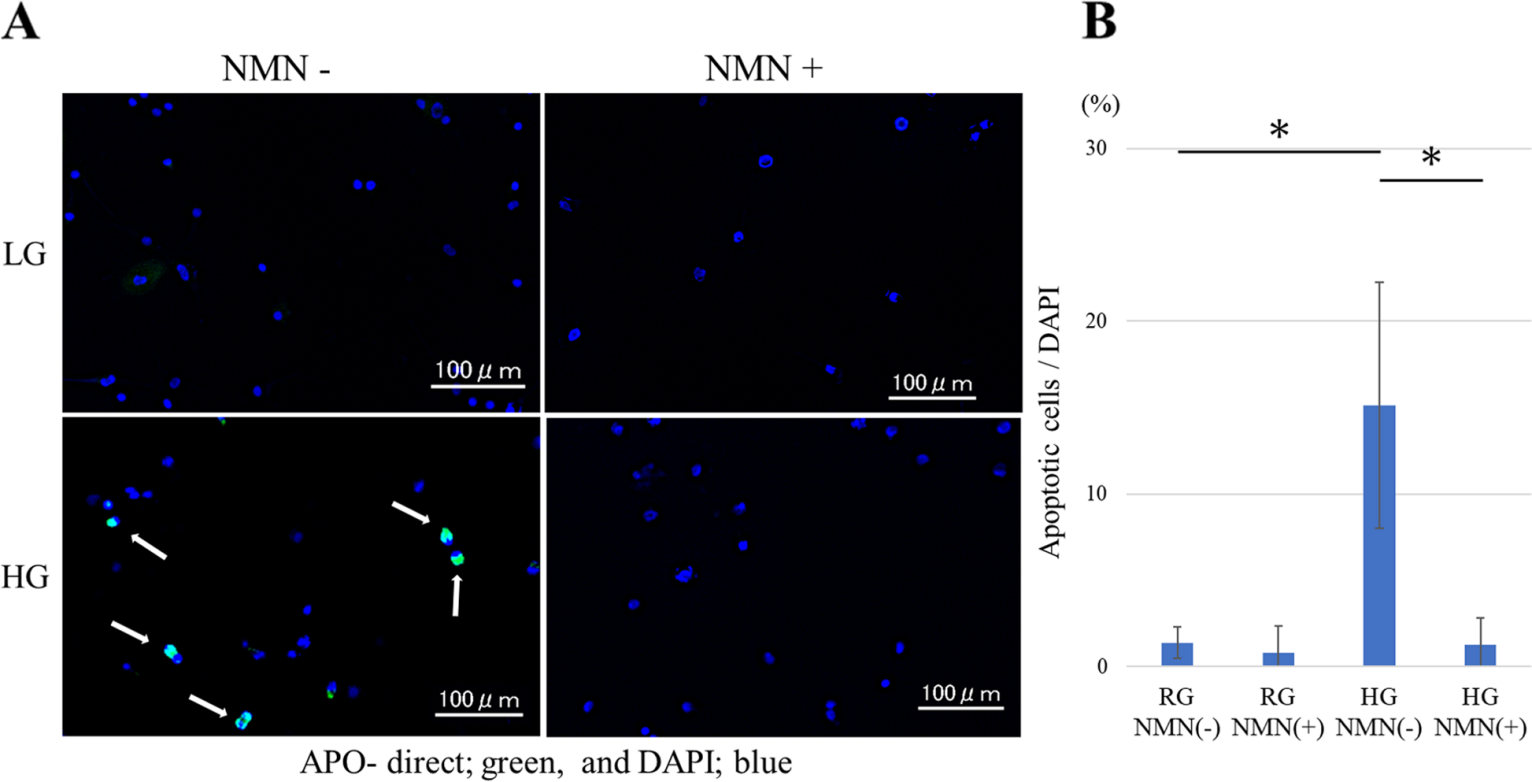
Analysis of cell apoptosis in vitro. Analysis of apoptotic markers in four different in vitro conditions: regular-glucose without NMN (RG NMN(−)), regular-glucose with NMN (RG NMN(+)), high-glucose without NMN (HG NMN(−)), and high-glucose with NMN (HG NMN(+)). (**a**) Fluorescence staining showing nuclear fragmentation in apoptotic tenocytes (green; white arrow) counterstained with DAPI (blue). (**b**) Quantification of apoptotic cells in the four groups. The number of apoptotic cells was significantly higher in the HG NMN(−) group than in the RG NMN(−) group, whereas that was significantly lower in the HG NMN(+) group than in the HG NMN(−) group. * *p* < 0.05

Fluorescence staining showed nuclear fragmentation in apoptotic cells (Fig. 5A). Based on counterstaining with DAPI, the number of apoptotic cells was significantly higher in the HG NMN(−) group than in the RG NMN(−) group at 48 h (*p* = 0.002) (Fig. 5B). However, the number of apoptotic cells was significantly lower in the HG NMN(+) group than in the HG NMN(−) group (*p* = 0.002); whereas no significant difference was detected between the RG groups (Fig. 5B).

### In vivo experiments

#### NOX immunostaining assessment

**Fig. 6:**
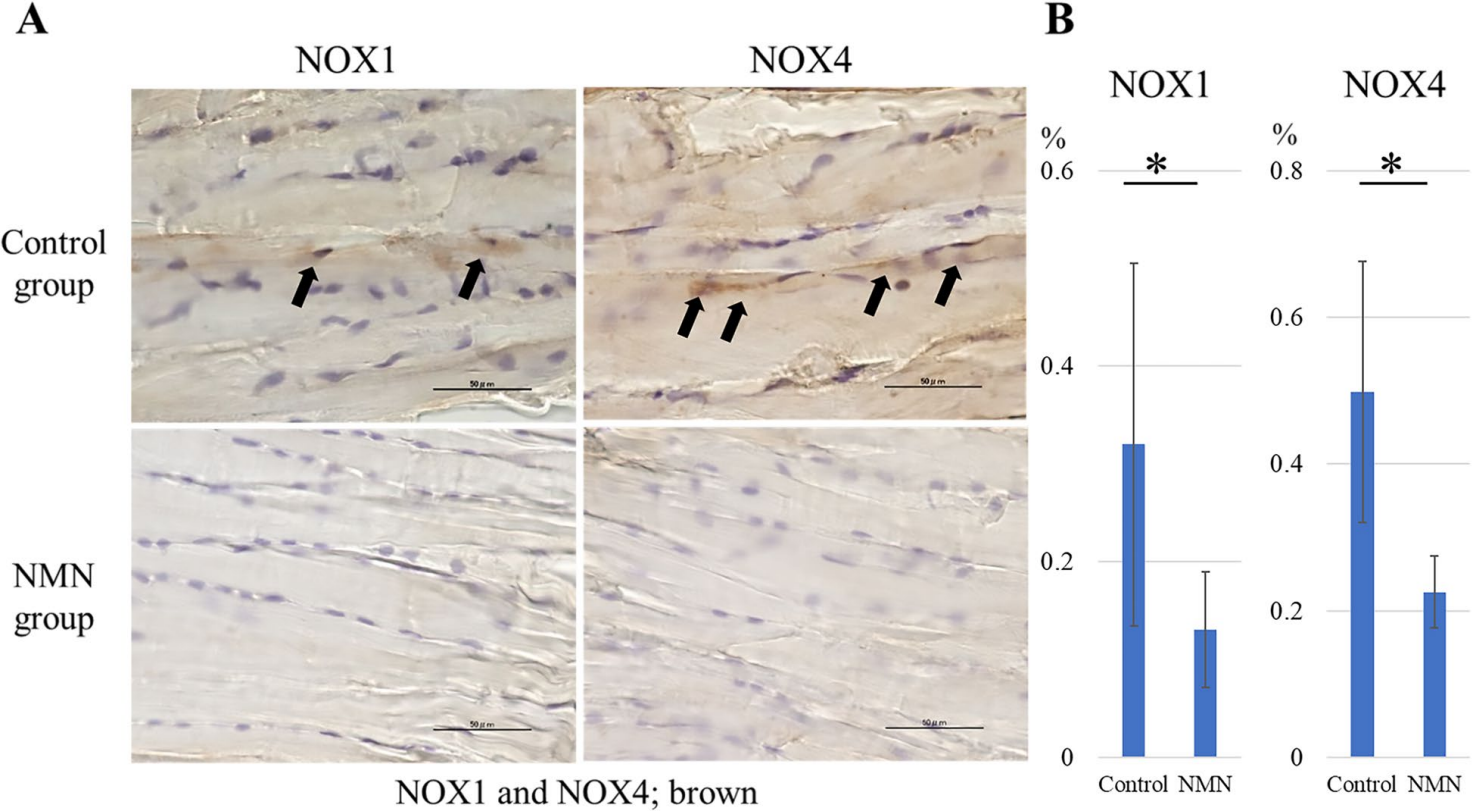
NOX immunostaining in vivo*.* NOX immunostaining in an in vivo collagenase-induced tendinopathy model. (**a**) NOX1 and NOX4 immunostaining (brown; black arrow) in the NMN group and control group. (**b**) Quantitative evaluation of NOX1 and NOX4 immunostaining. The levels of NOX1 and NOX4 were significantly suppressed in the NMN group compared to the control group. * *p* < 0.05

After 2 weeks of treatment, immunostaining revealed significantly suppressed NOX1 and NOX4 levels in the Achilles tendons of the NMN group compared to the control in a collagenase-induced tendinopathy model (*p* = 0.043 and *p* = 0.021, respectively) (Fig. 6).

#### SOD activity

**Fig. 7:**
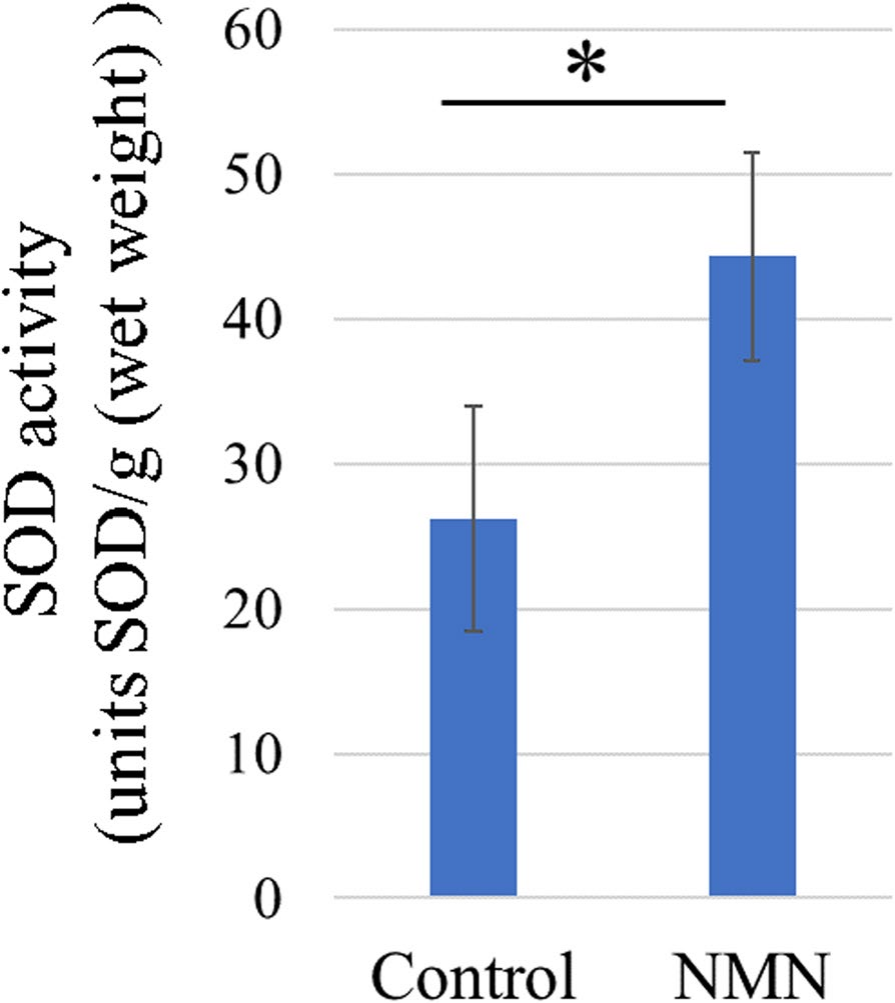
SOD activity measurement in vivo. SOD activity in an in vivo collagenase-induced tendinopathy model. SOD activity per wet weight of tendon is shown for the NMN group and control group. SOD activity was significantly higher in the NMN group than in the control group. * *p* < 0.05

After 2 weeks of treatment, SOD activity was significantly higher in the NMN group than in the control group in the Achilles tendons of a collagenase-induced tendinopathy model (*p* = 0.021) (Fig. 7).

#### Gene expression analysis by RT-PCR

**Fig. 8:**
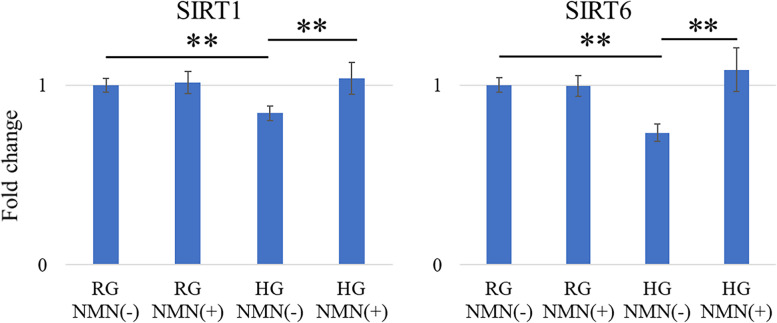
Quantitative expression of SIRT1, SIRT6, and IL6 mRNA in vivo. Quantitative RT-PCR results showing the mRNA expression of SIRT1, SIRT6, and IL6 in an in vivo collagenase-induced tendinopathy model. Expression is shown for the NMN group and the control group. The expression of IL6 was significantly suppressed in the NMN group compared to the control group, whereas the expression of SIRT1 and SIRT6 was significantly increased in the NMN group compared to the control group. * *p* < 0.05; ** *p* < 0.0001

The expression of IL6 in the Achilles tendon of the collagenase-induced tendinopathy model was significantly suppressed following 2 weeks of NMN treatment compared to the control group (*p* < 0.0001) (Fig. 8). In contrast, the expression of SIRT1 and SIRT6 was significantly increased in the NMN group compared to the control group (*p* < 0.0001 and *p* = 0.008, respectively) (Fig. 8).

## Discussion

Despite the elevated prevalence of tendinopathy [1, 2], its pathogenesis and mechanism of action are not fully understood, preventing the development of an effective treatment. Recently, it has become apparent that tendinopathy is associated with oxidative stress [9, 10, 31, 32]. Microinjuries associated with overload and repetitive mechanical stimulation induce excessive production of ROS in tendon cells [10, 31]. Oxidative stress was shown to inhibit tendon healing in a tendinopathy rat model in vivo [33], as well as block tenocyte differentiation in vitro [34]. Furthermore, continuous oxidative stress has been reported to induce apoptotic death in tenocytes through the activation of caspase-3 [35]. These studies indicate that alleviating oxidative stress in tendons may represent a promising therapeutic strategy for tendinopathy.

In the present study, the antioxidant potential of NMN was first evaluated in an oxidative stress model of tenocytes in vitro. Tendinopathy is common in diabetic patients [36], and a hyperglycemic load has been found to cause overproduction of ROS via increased NOX expression in tenocytes [11]. In addition, mitochondrial dysfunction caused by hyperglycemia is another inducer of apoptosis [37]. The present in vitro study shows that a hyperglycemic load upregulated NOX and IL6 expression, while also increasing ROS accumulation and apoptosis, indicating the successful establishment of an oxidative stress model in tenocytes.

NMN is a precursor of NAD^+^ and its administration has been shown to promote NAD^+^ biosynthesis [38], causing upregulation of key NAD^+^-consuming enzymes, such as sirtuins, poly (ADP) polymerase, and CD38/157 ectoenzyme, which play important roles in various biological processes [18, 39]. The present in vitro results demonstrate that NMN stimulates the cell proliferative capacity and prevents the downregulation of SIRT1 and SIRT6 in an oxidative stress tenocyte model. Reports on the involvement of high cellularity in tendinopathy are limited to histopathological findings, and there are no reports showing that tenocytes in tendinopathy models are highly proliferative in vitro. Furthermore, previous studies of tendinopathy models with hyperglycemia also support the present in vitro results [16, 27].

NMN has been found to inhibit ROS production and oxidative stress in the heart and brain [21, 22, 40]. However, to the best of our knowledge, this is the first report to evaluate the antioxidant effect of NMN in tendons. The antioxidant function of NMN has been linked to maintenance of mitochondrial homeostasis and increased levels of antioxidants [22, 40, 41]. Furthermore, SIRT1, which is stimulated by NMN, has been associated with suppressing oxidative stress by downregulating NOX production [42, 43]. As we have previously shown, inhibition of NOX1 and NOX4 blocked ROS production in rat tenocytes [16]. The present in vitro results confirm that NMN inhibits ROS accumulation by decreasing the expression of NOX1 and NOX4 in an oxidative stress tenocyte model.

Using an in vivo collagenase-induced tendinopathy model developed in 2019 by Hsiao et al. [34], the present study demonstrates that NMN administration downregulated NOX1 and NOX4 expression, while enhancing SIRT1 levels and SOD activation, resulting in decreased oxidative stress. Accordingly, the in vivo results confirmed the findings observed in vitro.

Previously, overproduction of ROS was shown to induce cellular damage and apoptosis by activating caspases and regulating the expression of the Bcl-2 family of proteins [44]. In addition, Fan et al. (2019) showed that SIRT6 reduced hyperglycemia-induced podocyte apoptosis by activating 5′ adenosine monophosphate protein kinase [45]. Here, in vitro results showed that administration of NMN blocked apoptosis by decreasing ROS levels, which was achieved through inhibition of NOX and activation of sirtuins.

This study has also some limitations. First, the in vitro monolayer culture of tenocytes does not adequately reproduce actual physiological conditions, although studies have suggested that primary tenocytes are phenotypically stable until passage 5 [46]. Second, the collagenase-induced tendinopathy model used in vivo resembles human acute tendinopathy and, therefore, differs from the pathogenesis of chronic tendinopathy commonly encountered in clinical practice. A model of chronic tendinopathy induced by repeated injections of collagenase at regular intervals needs to be developed and evaluated in future studies. Finally, the in vitro and in vivo oxidative stress models differ in the mechanism of oxidative stress induction: in vitro by hyperglycemia, and in vivo by collagenase type I injection. However, the in vivo evaluation in this study focused on investigating the effect of NMN on NOX expression and oxidative stress in the Achilles tendon, and both previous in vitro and in vivo models have been used to evaluate oxidative stress.

In conclusion, the in vitro and in vivo results described in this study show that NMN exerts an antioxidant effect on early tendinopathy by increasing the expression of sirtuins but inhibiting that of NOXs. These results suggest that NMN may be used as a potential treatment for tendinopathy.

## Data Availability

All data generated or analysed during this study are included in this published article.

## Abbreviations

DAPI: 2-(4-amidinophenyl)-1H-indole-6-carboxamidine
DMEM: Dulbecco’s modified Eagle’s medium
HG: High-glucose
IL: Interleukin
NAD^+^: Nicotinamide adenine dinucleotide
NMN: Nicotinamide mononucleotide
NOX: Nicotinamide adenine dinucleotide phosphate oxidase
PBS: Phosphate-buffered solution
RG: Regular-glucose
ROS: Reactive oxygen species
WST: Water-soluble tetrazolium salt
SIRT: Sirtuin
