# Correction: Antioxidant effect of nicotinamide mononucleotide in tendinopathy

**DOI:** 10.1186/s12891-022-05424-4

**Published:** 2022-06-06

**Authors:** Kohei Yamaura, Yutaka Mifune, Atsuyuki Inui, Hanako Nishimoto, Takashi Kurosawa, Shintaro Mukohara, Yuichi Hoshino, Takahiro Niikura, Ryosuke Kuroda

**Affiliations:** Department of Orthopaedic Surgery, Kobe University Graduate School of Medicine, 7-5-1, Kusunoki-cho, Chuo-ku, Kobe, 650-0017 Japan

**Correction: BMC Musculoskelet Disord 23, 249 (2022)**

**https://doi.org/10.1186/s12891-022-05205-z**

Following the publication of the original article [1] the author noticed that the published Fig. 8 is incorrect.

The original article [1] has been updated.

Below is the correct Fig. 8.

**Fig. 8:**
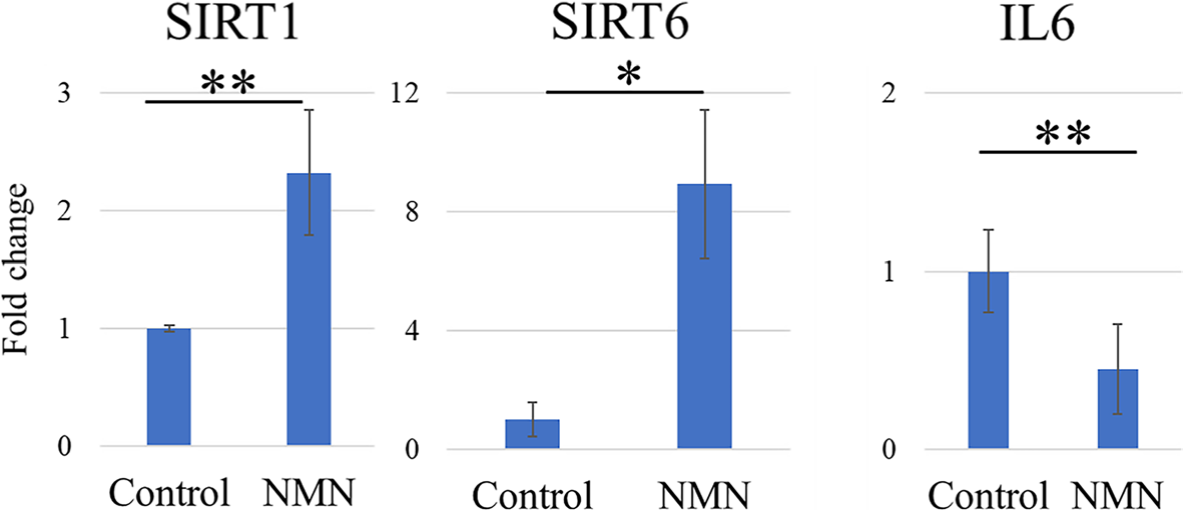
Quantitative expression of SIRT1, SIRT6, and IL6 mRNA in vivo. Quantitative RT-PCR results showing the mRNA expression of SIRT1, SIRT6, and IL6 in an in vivo collagenase-induced tendinopathy model. Expression is shown for the NMN group and the control group. The expression of IL6 was significantly suppressed in the NMN group compared to the control group, whereas the expression of SIRT1 and SIRT6 was significantly increased in the NMN group compared to the control group. * p < 0.05; ** p < 0.0001
